# Enhancing Antimicrobial Peptide Activity through Modifications of Charge, Hydrophobicity, and Structure

**DOI:** 10.3390/ijms251910821

**Published:** 2024-10-09

**Authors:** Przemysław Gagat, Michał Ostrówka, Anna Duda-Madej, Paweł Mackiewicz

**Affiliations:** 1 Faculty of Biotechnology, University of Wroclaw, Fryderyka Joliot-Curie 14a, 50-137 Wroclaw, Poland; michal.ostrowka2@uwr.edu.pl (M.O.); pawel.mackiewicz@uwr.edu.pl (P.M.); 2 Department of Microbiology, Faculty of Medicine, Wroclaw Medical University, Chalubinskiego 4, 50-368 Wroclaw, Poland; anna.duda-madej@umw.edu.pl

**Keywords:** antimicrobial peptides, antimicrobial resistance, cationic peptides, charge, hydrophobicity, peptide optimization, synthetic peptides

## Abstract

Antimicrobial peptides (AMPs) are emerging as a promising alternative to traditional antibiotics due to their ability to disturb bacterial membranes and/or their intracellular processes, offering a potential solution to the growing problem of antimicrobial resistance. AMP effectiveness is governed by factors such as net charge, hydrophobicity, and the ability to form amphipathic secondary structures. When properly balanced, these characteristics enable AMPs to selectively target bacterial membranes while sparing eukaryotic cells. This review focuses on the roles of positive charge, hydrophobicity, and structure in influencing AMP activity and toxicity, and explores strategies to optimize them for enhanced therapeutic potential. We highlight the delicate balance between these properties and how various modifications, including amino acid substitutions, peptide tagging, or lipid conjugation, can either enhance or impair AMP performance. Notably, an increase in these parameters does not always yield the best results; sometimes, a slight reduction in charge, hydrophobicity, or structural stability improves the overall AMP therapeutic potential. Understanding these complex interactions is key to developing AMPs with greater antimicrobial activity and reduced toxicity, making them viable candidates in the fight against antibiotic-resistant bacteria.

## 1. Introduction

The experiments conducted by scientists such as Robert Koch and Louis Pasteur provided compelling evidence for the role of microorganisms in causing infectious diseases, and thereby being behind the death of countless human beings [1]. The discovery of the first antibiotics, including penicillin by Alexander Fleming in 1928, and, more importantly, their mass production—penicillin from 1945—triggered a new era in medicine, transforming the treatment of bacterial infections. This period, known as the golden era of antibiotics, lasted until the 1970s and witnessed remarkable advancements with hardly any new classes of antibiotics discovered since then [2,3,4].

It is important to acknowledge that the golden era of antibiotics also marked the beginning of antimicrobial resistance (AMR). As the widespread use, and overuse, of antibiotics became commonplace in healthcare, agriculture, and animal breeding, bacteria started developing mechanisms to resist them, progressively reducing their effectiveness over time [4,5,6,7]. This phenomenon has been observed among both Gram-positive and Gram-negative bacteria. The combination of AMR with several other factors, including the scarcity of new antibiotics discovered and the dwindling interest of pharmaceutical companies in antimicrobial research, raises concerns that the 21st century might become a post-antibiotic era [7,8,9]. Accordingly, the World Health Organization has been treating AMR as a top global health problem, and also listed the priority pathogens to address [10,11,12]. In the first place, there are carbapenem-resistant non-fermenting rods of the genus *Acinetobacter* spp. and *Pseudomonas* spp., and the order *Enterobacterales* resistant to carbapenems and third-generation cephalosporins. Other highly and medium ranked positions on the list include the following: (i) *Staphylococcus aureus* methicillin-resistant (MRSA) and vancomycin-resistant (VRSA); (ii) vancomycin-resistant *Enterococcus* spp.; (iii) fluoroquinolone-resistant *Salmonella* spp., *Campylobacter* spp., and *Shigella* spp.; (iv) third-generation cephalosporin-resistant and fluoroquinolone-resistant strains of *Neisseria gonorrhoeae*; (v) clarithromycin-resistant strains of *Helicobacter pylori*; (vi) penicillin-resistant strains of *Streptococcus pneumoniae*; and (vii) ampicillin-resistant *Haemophilus influenzae*. These microorganisms are mostly associated with hospital environments, where the transfer of genes responsible for drug resistance is facilitated. As a result, they affect already vulnerable individuals, causing higher mortality among patients [12,13,14].

The post-antibiotic era means that healthcare would regress into a time when even a minor infection could prove fatal. Unfortunately, estimating the actual number of deaths worldwide due to AMR is challenging as most deaths go unregistered, and those that are registered often do not mention AMR because of the rules governing mortality statistics [15]. However, reports indicate that each year more than 60,000 people die because of AMR only in the European Union (including the European Economic Area) and the United States, and possibly as many as 700,000 globally. It means that AMR is among the leading causes of death in the world, and we are transitioning into the post-antibiotic era [16,17,18]. The gravity of the AMR crisis calls for immediate action and the development of new antimicrobial agents. Importantly, antimicrobial peptides (AMPs) are considered among the potential solutions [19,20].

**Figure 1:**
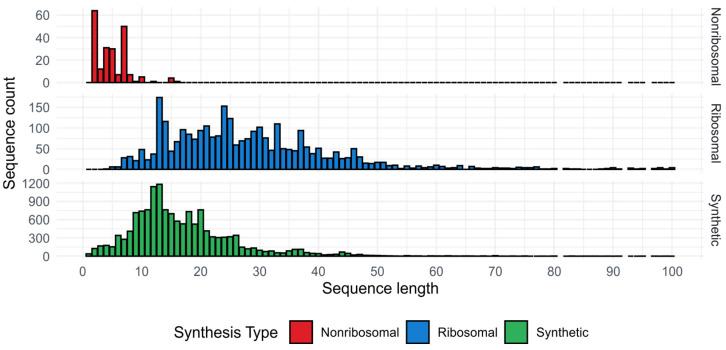
Distribution of peptide lengths for synthetic (14,434), ribosomal (2966), and non-ribosomal AMPs (213) based on data from DBAASP database (downloaded on 24 January 2024) [23]. We excluded sequences containing non-standard amino acids (4412 sequences) and longer than 100 residues (17 sequences).

AMPs represent a diverse group of peptides, generally fewer than 50 amino acids (Figure 1), but some proteins are also classified as AMPs if they exhibit the ability to kill or inhibit the growth of microorganisms. It is worth noting that the first discovered AMP was actually a small protein, called lysozyme, and it was isolated in 1922 by Alexander Fleming, predating his penicillin discovery by six years [21]. Importantly, the broad definition of AMPs makes their studying very challenging as there are no clear criteria for their identification, characterization, and classification. Some AMPs, especially those synthesized via non-ribosomal pathways, e.g., gramicidins, polymyxins, and bacitracin, are classified as antibiotics, more precisely peptide antibiotics [22]. The non-ribosomal AMPs are the shortest, usually smaller than 10 amino acids, whereas the length of most artificially synthetized AMPs ranges from 10 to 20 residues (Figure 1).

AMPs are characteristic of all living organisms and have been obtained from natural sources for decades, although most novel AMPs are now de novo synthetized. In multicellular organisms, AMPs play a crucial role as the initial defense against infectious microorganisms, e.g., bacteria, fungi, protists, and viruses [24,25,26]; however, they have also been shown to successfully target cancer cells, contribute to wound healing and angiogenesis, as well as exert immunomodulatory properties [27,28,29]. In turn, microorganisms use AMPs to combat other microorganisms for self-protection and competition [26,30,31].

**Figure 2:**
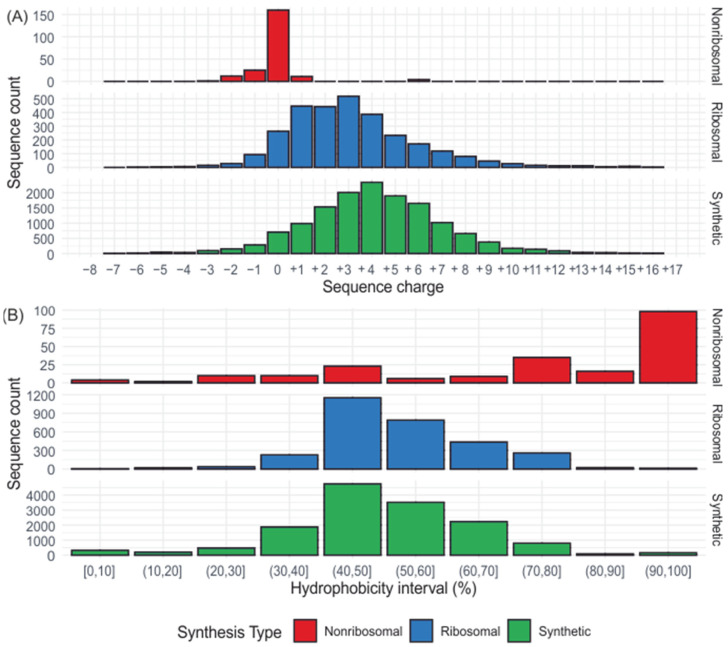
Distribution of synthetic (14,434), ribosomal (2966), and non-ribosomal (213) AMPs’ net charge (**A**) and hydrophobicity (**B**) based on data from DBAASP database (downloaded on 24 January 2024) [23]. The net charge of each AMP was calculated in R using the Peptides package. To identify the hydrophobic amino acids, we used the scale by Abraham and Leo [35]: alanine (Ala, A), cysteine (Cys, C), isoleucine (Ile, I), leucine (Leu, L), methionine (Met, M), phenylalanine (Phe, F), proline (Pro, P), tryptophan (Trp, W), tyrosine (Tyr, Y), valine (Val, V). Sequences containing non-standard amino acids (4412 sequences) were not included in the analysis.

**Figure 3:**
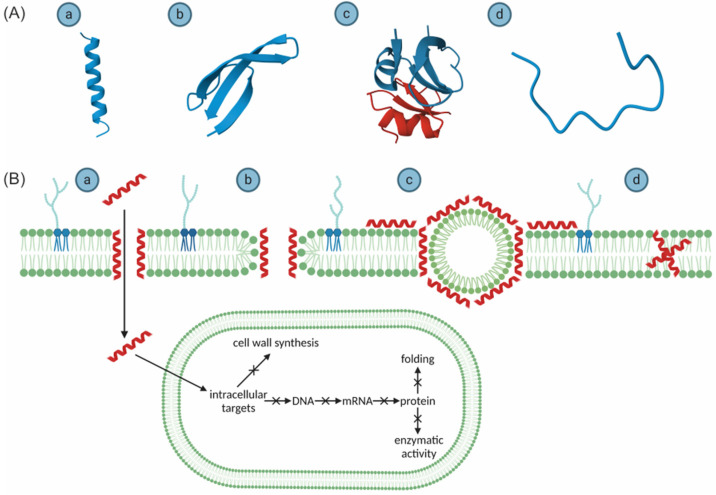
Structure types of AMPs and models of their antibacterial activity. Based on their structures, AMPs can be divided into four categories: (a) α-helical peptides, e.g., magainin-2 (PDB ID: 2MAG); (b) β-sheet peptides, e.g., human defensin 5 (PDB ID: 2LXZ); (c) peptides including both α-helix and β-sheet structure, e.g., human beta-defensin 2 (PDB ID: 1FD4); (d) linear peptides, e.g., indolicin (PDB ID: 1QXQ) (**A**). There are four main models of membrane pore formation: (a) barrel-stave model: peptides bind to bacterial membranes and assemble into a barrel-shaped cluster, creating pores; (b) toroidal model: accumulation of peptides prompts the lipid monolayers to undergo continuous curvature across the pore, which results in the formation of a water core that is lined by peptides and the lipid head groups; (c) carpet model: peptides cover the cell membrane like a carpet, disrupting the lipid bilayer similarly to detergents; (d) aggregate model: peptides bind to lipid head groups and randomly aggregate, creating channels for ion leakage across the membrane. After penetrating the membrane, AMPs can interact with bacterial intracellular molecules, inhibiting DNA, RNA, and protein synthesis, protein folding, protein enzymatic activity, and synthesis of cell wall components (**B**).

Most AMPs, especially ribosomal and synthetic, are rich in positive amino acid residues; only the non-ribosomal peptides are more balanced in terms of charge (Figure 2). Another characteristic feature of AMPs is the prevalence of hydrophobic amino acids. Ribosomal and synthetic AMPs demonstrate a similar distribution, whereas non-ribosomal peptides show a much greater excess of the hydrophobic residues, even more than 90% (Figure 2). All these common features do not have to correspond to similarities in their amino acid sequence. The proper placement of the positive and hydrophobic residues allows AMPs to fold into amphipathic secondary structures, exhibiting both water-soluble and water-insoluble regions, especially upon contact with the lipid bilayer. Considering their secondary structures, AMPs can be categorized as follows: (i) α-helical peptides, (ii) β-sheet peptides, (iii) peptides with α–β cross structures, and (iv) extended linear peptides featuring specific amino acids, e.g., glycine (Gly, G), tryptophan (Trp, W), and proline (Pro, P) (Figure 3) [32,33,34].

The positive charge, hydrophobicity, and amphipathy define the mode of action of AMPs. Firstly, due to their cationicity, AMPs are electrostatically attracted to the negatively-charged microbial or cancer cell membranes, but not the eukaryotic ones, which are zwitterionic (neutral) and additionally contain stabilizing cholesterol. This electrostatic interaction provides a certain level of selectivity, allowing AMPs to accumulate at bacterial membranes in higher concentrations than at eukaryotic cells, thereby favoring bacterial membrane disruption. Next, their hydrophobic segments enable them to penetrate the lipid bilayers and, in a concentration-dependent manner, disrupt the membrane by forming pores and/or micellization (Figure 3). The disruption of the cell membrane triggers cytoplasmic leakage and may lead to cell death by osmotic shock. This mode of action is non-specific, as AMPs interact with many various components of the bacterial cell [32,33,34]. This also gives them an advantage over traditional antibiotics, which specifically target a single enzyme, making AMPs less prone to AMR [36,37,38]. Alternative mechanisms of action include binding to specific cytosolic macromolecules, and thereby inhibiting the synthesis of proteins, nucleic acids, and elements of the cell wall (Figure 3) [39,40].

Despite the clear advantages of AMPs, e.g., broad spectrum of activity, high selectivity, relatively low toxicity, and low propensity to induce resistance, only a small fraction of AMPs reported so far have been able to successfully complete all phases of clinical trials and become accessible to patients. They include the following: Bacitracin, Polymyxin B and E (colistin), Tyrothricin, Gramicidin D, Gramicidin S, and Daptomycin [41]. However, the huge number of AMP-related articles and patents indicates that they have already attracted significant attention from the scientific community [41,42]. Finding efficient AMPs is not an easy task because only few AMPs with significant antimicrobial properties show effectiveness in laboratory experiments, and even fewer go through strict clinical trials successfully. In this review, we elaborate on the key properties conditioning AMP effectiveness, and how to boost them by applying appropriate modifications.

## 2. Optimization of AMPs

The effectiveness of AMPs hinges on a complex interplay of several interconnected characteristics that define their mode of action, such as the following: net charge, hydrophobicity, amphipathicity, and structural propensity. By carefully balancing these parameters, we can fine-tune AMPs. Over the past decades, researchers have been modifying and optimizing AMPs in this context, enhancing their therapeutic potential by increasing antimicrobial activity and reducing toxicity [43,44], and also deepening our understanding of their structure–function relationships [45,46]. Gained insights paved the way for a change in the AMP development strategy, moving from a single-factor modification to a more comprehensive de novo design [47]. This approach is implemented using theoretical knowledge and logic to generate short AMP sequences that are cheaper but still exhibit antimicrobial properties and selectivity. As a result, some public databases contain more synthetic than natural AMPs (Figure 1). The huge progress in AMP studies would not have been possible without bioinformatic tools that are vital for their prediction [48,49,50,51,52,53,54] and enable the analysis of their structural and functional properties [48,55,56,57,58,59,60].

### 2.1. Charge

Since cationic AMPs are electrostatically attracted to the negatively charged components of microbial membranes, increasing their net positive charge could enhance the interaction. This, in turn, would facilitate their aggregation at the bacterial membrane, allowing them to reach the threshold concentration required for membrane rupture. Consequently, the increase in charge should boost their antimicrobial activity [47]. The positive charge of AMPs is determined by the following: lysine (Lys, K, side-chain group pKa ~ 10.5), arginine (Arg, R, side-chain group pKa ~ 12.5), and, in slightly acidic conditions, histidine (His, H, side-chain group pKa ~ 6.0). Therefore, optimizing the number of these amino acids can influence the cationicity of AMPs. However, the optimal number of charged residues that truly enhance antimicrobial effectiveness and simultaneously minimize toxicity is not well defined.

To explore this topic, Bessalle et al. [61] synthesized magainin-2 (Figure 3 and its analogs, to which positively charged amino acid segments were added either to their N- or C-terminus. The addition of ten Lys or ten Arg residues increased magainin–2 antimicrobial activity from 8 to 18 times, depending on the bacterial strain, but not its hemolytic properties, i.e., a negative influence on erythrocytes. These basic residues also enhanced the ability to adopt α-helical structures in 50% trifluoroethanol (TFE), which mimics the membrane environment, but not in 50 mM potassium phosphate buffer. Importantly, amphipathic α-helices facilitate the AMP–lipid bilayer interaction, and thereby might have exerted some impact on the observed results in addition to the increased charge. However, the extension of the tag to 20 Lys residues, although resulting in higher antimicrobial activity, comparable to that of its ten-Lys analog, additionally augmented the hemolysis, making it unsafe for application in humans.

In another study, Higgs et al. [62] demonstrated that the antimicrobial activity of chicken avian β-defensin-8 can be raised through replacing the hydrophobic valine (Val, V) and isoleucine (Ile, I) with basic Arg, or decreased through replacing these residues with negatively charged aspartic acid (Asp, D). Jiang et al. [63] provided further insight into the issue by altering the net positive charge +7 of the α-helic V13K peptide. V13K analogs lost both antimicrobial and hemolytic activity when the net charge was below +4. By increasing the net charge to +8, the authors enhanced its antimicrobial activity on average by 1.6 times without affecting its hemolytic properties. However, a net charge of +9 and +10 not only increased the antimicrobial activity of V13K but also dramatically boosted its hemolytic activity as well.

As demonstrated in various studies, the strategic manipulation of the AMP charge can optimize AMPs’ cationicity and potentially improve their effectiveness against microbial pathogens. The distribution of net charge for synthetic and ribosomal AMPs in Figure 2 indicates that this strategy is widely applied in designing synthetic peptides. However, the studies also reveal that there is a delicate balance when manipulating charge between increasing antimicrobial activity and AMP toxicity, and that the other major factors affecting AMP properties play a role in the process too. The disturbance of this balance can easily occur in short peptides, which are usually synthetic peptides (Figure 1).

### 2.2. Hydrophobicity

Another crucial factor which can influence the activity of AMPs is hydrophobicity. In general, it is defined as the percentage of hydrophobic residues in the peptide, which can facilitate the location of AMPs in the hydrophobic core of the plasma membrane [64,65]. About 80% ribosomal and 75% synthetic AMPs from the DBAASP database (dataset downloaded on 24 January 2024) [23] contained 40% to 70% hydrophobic residues, according to the hydrophobicity scale by Abraham and Leo [35] (Figure 2). This suggests that the hydrophobicity and ‘antimicrobialness’ of AMPs are interrelated. Accordingly, by raising this parameter, we could improve peptide antimicrobial activity [66]. However, the manipulation of hydrophobicity may also cause a decrease in antibacterial activity and an increase in toxicity towards eukaryotic cells as in the case of charge, because the highly hydrophobic peptides, due to their poor solubility, tend to bind to eukaryotic membranes and also trigger their disruption [67,68].

One of the approaches to enhance AMP effectiveness while preserving selectivity is to add hydrophobic residues at the end of the peptide. Various hydrophobic amino acids can serve as end-tags, but Trp and phenylalanine (Phe, F) are especially noteworthy. Both residues are aromatic and have a tendency to locate at the membrane–water interface, near the head group of phospholipids, thereby serving as anchors for AMPs [69,70,71,72,73].

Schmidtchen et al. [74,75] performed a series of studies in which they investigated how effective Trp and Phe are in increasing antimicrobial properties. Firstly, the minimal inhibitory concentration (MIC) of GKH17, a peptide derived from kininogen, was significantly lowered by Trp-Trp-Trp tagging on the C- or N-terminus against *S. aureus*, maintaining low cytotoxicity against eukaryotic cells. However, a further increase in the tag length to five Trp residues did not result in higher antimicrobial activity but increased its hemolytic effect. In turn, Phe-Phe-Phe-Phe-tagged GKH17 (and its further extension to five Phe residues) boosted the antimicrobial activity of the peptide with only a slight increase in its hemolytic properties, which was, however, still lower than the hemolytic effect of LL–37—a benchmark peptide [74]. In their next studies, Schmidtchen et al. [75] tested GRP10, a ten amino acid-long peptide derived from proline/arginine-rich end leucine-rich repeat protein (*abbr.* PRELP). They found that GRP10 alone was not effective against the *Escherichia coli*, *Pseudomonas aeruginosa*, and *S. aureus* ATTC strains, but GRP10W4N (four Trp-tagged N-terminal analog) did exhibit strong antimicrobial and low hemolytic activity. Similar results were obtained by Malmsten et al. [76], who expanded the number of tested peptide analogs derived from PRELP. Their overall experiments indicate that both Trp/Phe-tagged peptides in general showed increased antimicrobial properties; however, when the tag length exceeded four Trp/Phe amino acids, the hemolytic activity also increased.

There are some examples of research applying single amino-acid substitutions to manipulate AMP hydrophobicity, e.g., Molhoek et al. [77]. They designed several different analogs of the α-helical N-terminal segment (C1-15) of the chicken host defense peptide cathelicidin-2 (CATH-2) that exhibited a strong killing effect against both Gram-positive and Gram-negative bacteria at low cytotoxicity [78]. In their analogs, they substituted Phe residues, on the non-polar face of the peptides, for Trp and tested their ‘antimicrobialness’ in comparison to C1–15. The F2,5,12W variant (substitution at positions 2, 5, and 12) turned out to be the most effective against the tested bacteria, but also significantly more cytotoxic than C1–15 [77].

Another way of AMP modification to influence peptide hydrophobicity is to conjugate them with cholesterol or fatty acids. Chen et al. [79] applied N-terminal cholesterol modification to PMAP-37 (F34-R), a peptide derived from porcine myeloma cells (with Phe-to-Arg substitution), developing a novel Chol-37 (F34-R) peptide. In PMAP-37 (F34-R), the substitution alone affected the peptide charge (from +9 to +10) and structure, slightly improving amphipathy and increasing PMAP-37 antibacterial activity with no apparent hemolytic effect toward erythrocytes [80]. Importantly, Chol-37 (F34-R) exhibited even stronger antimicrobial and antibiofilm activity compared to PMAP-37 (F34-R), maintaining low cytotoxicity against eukaryotic cells. In addition, cholesterol made it more stable in different pH, salt, and serum environments [79]. On the other hand, research on anoplin analogs showed that the conjugation of longer (12–14 carbons) fatty acids, but not shorter ones, resulted in better antimicrobial activity; however, they also triggered higher toxicity in eukaryotic cells [81,82]. Similar results were obtained for bovine lactoferricin fragments conjugated with linoleic acid, i.e., this modification improved antimicrobial activity but at the cost of increased toxicity [83].

The last modification described in this subchapter concerns N–methylation, which can be achieved through the methylation of the backbone amides or methylation of the side chains of some amino acids. Smirnova et al. [84] synthesized 45 analogs of indolicidin, isolated from the cytoplasmic granules of the bovine neutrophils [85]. The most potent one, ‘analogue 34’, contained all Trp residues substituted for (2–Me)Phe. Due to the modification, they observed 3-fold more antimicrobial and at least 1.8-fold less hemolytic activity—the analog reached the detection limit—compared to the indolicidin.

### 2.3. Structure

The propensity to form secondary amphipathic structures is another key factor that conditions the ‘antimicrobialness’ of AMPs. In aqueous solution, many α-helical AMPs are primarily disordered, but upon interaction with biological membranes, they adopt an appropriate secondary structure [86,87]. Conversely, β-sheet peptides do not undergo such drastic changes but stay rigid in aqueous solutions to avoid the exposure of their hydrophobic facet and potential multimerization [86].

Importantly, a stable amphipathic α-helical structure is not only an effective weapon against microorganisms but also affects eukaryotic membranes. An example of this is the α-helical peptide melittin, the major and most potent component of the venom of *Apis mellifera*, which has both high antimicrobial and hemolytic activity [88]. To mitigate the latter effect, Asthana et al. [89] designed two melittin analogs, in which leucine (Leu, L) was substituted for alanine (Ala, A) at position 6 (MM–1), and both 6 and 13 (MM–2). Previously, they discovered that the melittin hemolytic activity is potentially caused by a leucine zipper motif formed by these Leu residues. The tested analogs showed a similar antimicrobial activity, but MM-1 exhibited up to 10 times lower and MM-2 up to 100 times lower hemolytic activity compared to the wild-type melittin. The membrane permeability experiments and the determination of the secondary structures of MM-1, MM-2, and melittin indicated that the analogs indeed exhibited fewer abilities to disrupt the membranes and form α-helical structures in the presence of zwitterionic vesicles, but acted similarly to melittin in the presence of negatively charged membranes [89]. Importantly, melittin interacts with zwitterionic membranes adopting a pore-forming mechanism (the barrel-stave model) and with bacterial ones acting as a detergent (the carpet model) (Figure 3). Asthana et al. [89] suggested that the leucine-to-alanine substitutions possibly disrupted only the former but not the latter mechanism.

Further studies demonstrated that the substitution of residues from the non-polar side of a peptide for positively charged amino acids, which disrupts the α-helical amphipathic structure, can decrease AMP hemolytic but not antimicrobial activity [90,91]. Such reports pushed Zhang et al. [92] to design AR–23 (melittin-related peptide of length 23) analogs, through the substitution of Ala1, Ala8, and Ile17 for Arg or Lys [92]. These changes indeed led to the following: (i) a decrease in the α-helical content of the analogs compared to AR–23, especially those having position 17 mutated, which is located at the peptide non-polar side, and (ii) the reduction in their hemolytic/cytotoxic activity. The analog with the amino acid substitutions of Arg1, Arg8, and Lys17-A (A1R, A8R, I17K) exhibited the best therapeutic potential (measured as the ratio of hemolytic activity and MIC) but was not effective against *S. aureus*. Importantly, these mutations not only affected the analogs’ structure but also charge and hydrophobicity. In the case of A (A1R, A8R, I17K), a decrease in helicity and hydrophobicity was observed but also an increase in amphipathy and positive charge. As in the case of melittin analogs MM–1 and MM–2, A (A1R, A8R, I17K) did not bind/penetrate eukaryotic membranes but exhibited increased binding/penetration to *E. coli* membranes, as evidenced by Propidium iodide staining assay. It seems that the Lys in the center of the non-polar face of the peptide keeps it unstructured in benign water/physiological conditions but still allows it to assume α-helix in the hydrophobic environment, especially upon contact with bacterial membranes.

Interesting insights into AMP structure were presented by Srivastava et al. [93] using Temporin L (TempL), a 13-amino acid antimicrobial peptide isolated from the European common frog. They substituted the Phe residues at positions 5 and 8, which form a phenylalanine zipper, for either Ala or Leu. Both substitutions resulted in a decrease in TempL antimicrobial properties; however, their effects on peptide structure and other properties varied. In the case of the Leu substitutions, there was an increase in peptide helicity, hydrophobicity, and hemolytic/toxic properties, indicating that the leucine zipper stabilizes the structure but reduces selectivity for bacterial membranes, potentially explaining the decrease in antimicrobial activity. Notably, the selectivity was improved with a single Leu substitution in positions 5 or 8 because the hemolytic/toxic properties dropped and antimicrobialness was still maintained. Conversely, the Ala substitutions led to a decrease in helicity, hydrophobicity, and hemolytic/toxic properties, suggesting that Ala weakens the structural integrity of the peptide, impairing its ability to disrupt bacterial membranes.

In another study, Song et al. [94] systematically investigated the effect of end-tagging the Trp-rich antimicrobial peptide W4 (sequence: RWRWWWRWR-NH2) with various single hydrophobic residues at the C-terminus, namely Trp, Phe, Val, Ile, Leu, and Ala. They found that only the Ala-tagged W4 analog exhibited similar antibacterial activity to W4 while preserving low hemolytic properties. They suggested that this similarity was due to its better helicity compared to the other investigated W4 analogs. Moreover, the addition of Gly at the N-terminus of the Ala-tagged W4 further increased its helicity and slightly improved its antimicrobial potential, supporting the authors’ reasoning. Importantly, the Ala-tagged W4 was also less hydrophobic than the other investigated analogs, and the Gly- and Ala-tagged W4 was even less so, implying that the differences in hydrophobicity might also contribute to the improved selectivity compared to the other analogs; the other analogs might be simply too hydrophobic.

In addition to the intensive research on the role of α-helical structure in AMPs, there have also been some studies on the β-sheet peptides. They owe their rigid conformation to the hydrogen bonding of backbone atoms (the amide hydrogen and carbonyl oxygen) formed by the adjacent strands of the β-sheet. Li et al. [95] methylated the bonded amides in gramicidin S, disrupting them and reducing the peptide structural order. They designed 12 N-methylated gramicidin S analogs (NMe–GS) from which NMe-8 (Leu5→MeLeu, Pro7→MeAla) was the most promising. NMe–8 exhibited similar antimicrobial and five times reduced hemolytic activity to those of gramicidin S, while the other investigated N-methylated analogs were comparable or had lowered hemolytic properties. Importantly, methylation has also been reported to increase the toxicity of cyclic β-sheet peptides, and likely as a result of their increased hydrophobicity [96]. What is more, in the case of Protegrin–I (PG1), a β-hairpin AMP derived from porcine leukocytes [97,98,99], the stabilization of the β-hairpin conformation rather than its disruption was crucial to improve the peptide’s therapeutic potential. PG1 contains two disulphide bridges, the elimination of which (one or both of them) results in the loss of its activity [98]. Tan et al. [100] developed several backbone-cyclized analogs containing a varying number of disulphide bridges within the sequence. The most potent one, ccPG 3, contained three evenly spaced, parallel disulphide bonds, and exhibited significantly decreased hemolytic activity while preserving the antimicrobial activity against most (but not all) of the tested bacteria compared to PG1.

### 2.4. De Novo Design

The de novo design of AMPs involves carefully considering various AMP descriptors that altogether can be reduced to the abovementioned features, i.e., charge, hydrophobicity, and structure. These features can be balanced using a template design or machine learning-based design. Notably, the boundary between both approaches is not strict as templates are often generated and/or optimized using machine learning.

The template design typically employs specific α-helical amphipathic peptides with alternating hydrophobic and basic residues to enhance AMPs’ ability to interact with microbial membranes. For example, Wiradharma et al. [101] designed an α-helical template with a general sequence of (XXYY)_n_, where X represents a hydrophobic residue, Y represents a cationic residue, and n represents the number of repeat units. Out of the ten peptides they generated using this template, three ((FFRR)_3_, (LLRR)_3_, and (LLKK)_3_) had potent antimicrobial activity against *Bacillus subtilis* and low hemolytic activity against rat erythrocytes. Moreover, they observed that although the increase in the number of template units to four raised the peptides’ antimicrobialness, it also dramatically boosted their hemolytic properties. Further studies on this template produced an analog G (IIKK)_3_I–NH_2_, designed by Hu et al. [102], which showed strong activity against *E. coli* and *B. subtilis*, while also exhibiting low hemolytic activity [103]. As in the case of Wiradharma et al. [101], four IIKK repeats significantly enhanced both antimicrobial and hemolytic properties. Importantly, the peptides by Wiradharma et al. [101] and Hu et al. [102] with four repeat units were also more prone to forming α-helical structures and yielded more typical α-helical CD spectra compared to shorter sequences. This likely contributed to the loss of specificity observed with the four repeat units.

Another commonly used template is a heptad repeat sequence (abcdefg)n observed in α-helical structures, e.g., coiled coils. It is common in natural proteins, as well as AMPs such as melittin, BMAP–28, BMAP–27, and Piscidin–1 [89,104,105,106]. The key features of this template are as follows: position a and d occupied by hydrophobic residues, and position e and g filled by charged amino acids [107,108]. Based on this sequence pattern, Dou et al. [109] designed their own template (abcdefg)n–(W)n–(gfedcba)n, where the positions a and d represent Leu, Val, Phe, or Trp; W is the Trp linker; the other positions are occupied by Arg residues; and n represents the number of repeat units (from one to three). The most potent peptide they investigated was LW3 (LRRLRRR–WWW–RRRLRRL–NH2), and it was very effective against the ATCC strains of *E*. *coli*, *S*. *aureus,* and both the ATCC and drug-resistant strains of *P*. *aeruginosa*, and exhibited low hemolytic properties. Importantly, LW3 had a more stable α-helix and higher hydrophobicity compared to the other peptides that Dou et al. [109] investigated, but was less amphipathic, which again emphasizes how important the balance of these key characteristics is and not their maximization.

The de novo design of β-sheet antimicrobial peptides is not well studied compared to the α-helical AMPs [110]. To fill this gap, Shao et al. [111] designed the simplest β-hairpin AMP template RWYXYZZRWYXY–NH2, which consists of two hydrogen-bonded β-strands containing cationic (X = Arg, Lys, His) and hydrophobic residues (Y = Val, Ile, Leu, Phe) connected by a rigid turn region (ZZ = Pro–Gly, D–Pro–Gly, Asn–Gly). From the 24 analogs they designed, WKF–PG (RWFKFPGRWFKF–NH2) and WRF–NG (RWFRFNGRWFRF–NH2) exhibited significant bactericidal and anti-inflammatory effects in vitro and in vivo without observed toxicity. The superior performance of WKF-PG and WRF-NG comes from a combination of key features: a balanced charge and hydrophobicity, a stable structure, strong interactions between charged and aromatic parts of the peptides, and specific structural turns that boost their antimicrobial effectiveness while reducing toxicity.

In the context of a computational peptide de novo design, machine learning is not restricted to machine learning-assisted template design, but it also involves optimizing sequences through evolutionary algorithms and fitness functions, as well as predicting their antimicrobial properties. Evolutionary and genetic algorithms are especially noteworthy. They simulate the process of molecular evolution by generating new peptide sequences and assess their antimicrobial effectiveness through fitness functions. The latter quantify the suitability of a peptide based on specific properties such as charge, hydrophobicity, amphipathy, and activity against bacteria [112,113]. This approach has already been used to improve the therapeutic potential of the lipopolysaccharide-binding domain [114] or Temporin-Ali [115] and optimize the length of the hybrid α-helical peptide Cecropin–Melittin [116].

In addition to evolutionary algorithms, both traditional machine learning models (e.g., Support Vector Machines, Random Forests, and k-Nearest Neighbors) and deep learning models are widely used in de novo peptide design. Traditional models assess the peptide antimicrobial potential by analyzing key features, such as sequence composition, structural properties, or motifs, while deep learning models automatically extract relevant features and model complex relationships within the data. Both approaches allow to sift quickly and cost-effectively through large peptide databases and select candidates with desirable properties for in vitro and in vivo studies [50,117].

## 3. Application of AMPs

Importantly, the emerging field of AMP application to combat AMR faces more challenges than AMP optimization in terms of antimicrobial properties and toxicity. In order to become a real alternative to antibiotics, researchers have to address their high cost of production, thereby finding (i) new innovative methods of production and purification, (ii) more efficient substrates for overexpression, and (iii) optimal autoinduction strategies [118,119,120]. AMP metabolic stability is also a significant issue. To combat proteolytic degradation, the substitution of L- for D-amino acids [121,122,123], cyclization [124,125], and all-hydrocarbon stapling [126,127], including lysine tethering [128], have already been applied. High salt concentrations in human body fluids have also been addressed, e.g., by AMP conjugation with cholesterol [79], stabilization of their secondary structure using helix-capping [129], or substitution of tryptophan/histidine for β-naphthylalanine and β-(4,4′-biphenyl)alanine [130]. All these modifications not only improve metabolic stability but affect the overall peptide structure, hydrophobicity, amphipathy, and charge. For example, cyclization, which typically stabilizes the peptide by restricting its flexibility, might affect its amphipathy and hydrophobicity, and accordingly, interactions with bacterial membranes [131]. D-amino acids not only make peptides inaccessible for proteases but also alter peptide backbone conformation, which can influence the spatial arrangement of hydrophilic and hydrophobic residues and, accordingly, antimicrobial potential [132]. Non-natural amino acids are perhaps especially interesting for peptide optimization as they provide immense possibilities to fine-tune their properties and also possible problems with production [133].

It is also worth mentioning that AMPs are not only investigated as an alternative to traditional antibiotics but can be administered in combination with them, and together both have been demonstrated to display enhanced effectiveness compared to when they are used alone. The most common synergistic mechanism involves the ability of AMPs to facilitate the uptake of antibiotics by bacteria. The bacterial cell membrane is selectively permeable to various drugs but AMPs can alter the permeability, enabling the penetration of antibiotics into the cell. This allows antibiotics to reach and interact with their intracellular targets more effectively [134]. The therapy has shown promising results in combating multidrug-resistant bacteria, where conventional antibiotics alone may be less effective due to AMR (e.g., [135,136]). Additionally, AMPs can act as antibiotic adjuvants, e.g., the linear short AMP SLAP-S25 was shown to enhance the efficacy of multiple antibiotics, inhibit the growth of MDR Gram-negative bacteria, reverse antibiotic resistance, and reduce both the dosage and associated side effects of antibiotics [137].

## 4. Conclusions

The optimization of AMPs requires a delicate balance between various structural and functional parameters. The studies discussed above highlight the significance of factors such as charge, hydrophobicity, amphipathy, and structure in determining the effectiveness and safety of AMPs. By strategically manipulating these parameters, researchers have made substantial progress in understanding and enhancing the therapeutic potential of AMPs, i.e., balancing their antimicrobial activity and toxicity.

However, the optimization process of AMPs is not without its challenges. Increasing the positive charge, hydrophobicity, or stabilizing the amphipathic α-helical structure of AMPs can usually improve their interaction with bacterial membranes but also with eukaryotic ones; the same structural features are responsible for both interactions. The key issue is to manipulate the AMPs in the context of the differences in bacterial and eukaryotic membrane composition and/or using the differences in the AMP mode of action on bacterial vs. eukaryotic membranes, so that the former interaction is more affected, whereas the latter is less [89,138]. Importantly, using a scanning electron microscope and fluorescence microscopy, we can observe peptide–membrane interaction, and deduce the affinity for different membrane environments [139,140]. It is also apparent that sometimes it is better to slightly destabilize the secondary structure and lower the hydrophobicity or positive charge to achieve better peptide therapeutic potential [92]. Importantly, a strategic manipulation of one parameter—whether it is charge, hydrophobicity, amphipathy, or structure—could simultaneously affect the other key parameters, so the observable outcome might represent a synergistic effect of many small changes that together produce a desired or undesired effect. Unfortunately, studies often focus on checking only a single factor, e.g., charge, and do not investigate how it affects the structure, hydrophobicity, or amphipathy. Therefore, it is desirable to perform more comprehensive studies involving the modification of many peptide parameters. Such data could significantly improve the prediction and de novo design of AMPs [60,141,142].
